# Integrating multimodal imaging and peritumoral features for enhanced prostate cancer diagnosis: A machine learning approach

**DOI:** 10.1371/journal.pone.0323752

**Published:** 2025-05-15

**Authors:** Huadi Zhou, Mei Xie, Hemiao Shi, Chenhan Shou, Meng Tang, Yue Zhang, Yue Hu, Xiao Liu

**Affiliations:** 1 Department of Radiology, Zhejiang Hospital, Hangzhou, Zhejiang Province, China; 2 Department of Pathology, Zhejiang Hospital, Hangzhou, Zhejiang Province, China; 3 Guang’anmen Hospital, China Academy of Chinese Medical Sciences, Beijing, China; Memorial Sloan Kettering Cancer Center, UNITED STATES OF AMERICA

## Abstract

**Background:**

Prostate cancer is a common malignancy in men, and accurately distinguishing between benign and malignant nodules at an early stage is crucial for optimizing treatment. Multimodal imaging (such as ADC and T2) plays an important role in the diagnosis of prostate cancer, but effectively combining these imaging features for accurate classification remains a challenge.

**Methods:**

This retrospective study included MRI data from 199 prostate cancer patients. Radiomic features from both the tumor and peritumoral regions were extracted, and a random forest model was used to select the most contributive features for classification. Three machine learning models—Random Forest, XGBoost, and Extra Trees—were then constructed and trained on four different feature combinations (tumor ADC, tumor T2, tumor ADC+T2, and tumor + peritumoral ADC+T2).

**Results:**

The model incorporating multimodal imaging features and peritumoral characteristics showed superior classification performance. The Extra Trees model outperformed the others across all feature combinations, particularly in the tumor + peritumoral ADC+T2 group, where the AUC reached 0.729. The AUC values for the other combinations also exceeded 0.65. While the Random Forest and XGBoost models performed slightly lower, they still demonstrated strong classification abilities, with AUCs ranging from 0.63 to 0.72. SHAP analysis revealed that key features, such as tumor texture and peritumoral gray-level features, significantly contributed to the model’s classification decisions.

**Conclusion:**

The combination of multimodal imaging data with peritumoral features moderately improved the accuracy of prostate cancer classification. This model provides a non-invasive and effective diagnostic tool for clinical use and supports future personalized treatment decisions.

## Introduction

Prostate cancer is one of the most common cancers in men and the third leading cause of cancer-related death in men [1]. The prognosis of prostate cancer depends on various factors, including tumor stage, grade, patient age, and health status. Treatment may also cause a series of side effects, such as sexual dysfunction, urinary incontinence, and bowel dysfunction, severely affecting the quality of life [2].

Currently, the most common method of diagnosing prostate cancer typically follows serum prostate-specific antigen (PSA) testing or digital rectal examination (DRE), and succeeded by transrectal ultrasound (TRUS)-guided prostate biopsy. While prostate serum PSA screening has improved marginally in lowering the risk of death due to prostate cancer over a decade in a decade [3], its lack of specificity and sensitivity to factors such as age reduces its effectiveness as an indicator [4]. Therefore, there appears a greater probability of overdiagnosis and overtreatment due to false-positive elevations in serum PSA levels [5]. Moreover, DRE is commonly employed in clinical screening for the detection of perceivable masses and is highly dependent on the skill level of the physician [6], which leads to missed cases of prostate cancer as the physician is only able to evaluate the prostate’s peripheral zone. Research demonstrates that up to 30% of the time the decision of whether to pursue a TRUS guided biopsy or not is associated with the absence of diagnosed clinical prostate cancer [7]. Therefore, there appears necessity for more effective diagnostic processes for prostate cancer to avoid overtreatment of clinically low-risk cases and missing the diagnosis in clinically early-stage cases.

Due to the advancement of imaging methods, radiological screening methods are showcasing increased benefits in areas such as multiparametric magnetic resonance imaging (mpMRI) of the prostate as a novel diagnostic tool for prostate cancer. Research demonstrates that there appears a strong relationship between the changing tumor size and abnormal mpMRI findings, which are positively correlated with increased tumor volume and higher tumor grade [8]. Introducing this examination method into clinical diagnosis may reduce early missed diagnoses and overtreatment; while optimizing and developing mpMRI sequences offer previously unavailable cancer information for clinical practice, such as tumor volume, location, and lesion characteristics.

Four sequences typically constitute conventional mpMRI protocols: T1-weighted imaging (T1WI), T2-weighted imaging (T2WI), diffusion-weighted imaging (DWI), and dynamic contrast-enhanced imaging (DCE) [9].

First, the assessment of hemorrhage, the observation of anatomical structures, and the evaluation of lymph node metastasis are the key applications of T1WI in prostate MRI. Nevertheless, prostate cancer lesion detection is limited in value by this sequence, as prostate cancer typically presents with a signal intensity on T1WI that is similar to normal prostate tissue, rendering direct differentiation challenging [10].

The zonal anatomy of the prostate and its spatial relationship with the prostatic capsule can be visualized utilizing T2WI. Prostate volume measurement is facilitated by this sequence, and it also facilitates determining lesion location, dimensions, and extent, alongside the assessment of local invasion. Due to its high glandular ductal tissue content and high water content, the peripheral zone (PZ) of normal prostate tissue exhibits uniform high signal intensity on T2WI; whereas, prostate cancer, represented by high cellularity and reduced water content, appears as low signal intensity on T2WI. Moreover, a positive correlation exists between the degree of signal change and the aggressiveness of the cancer [11]. The transition zone (TZ), owing to the proliferation of prostatic epithelium and stroma, frequently displays heterogeneous low to intermediate signal intensity, resulting from high cell density and thus uneven signal intensity [12]. Subtle signal changes on T2WI, easily obscured by the complex signal intensity of the transition zone, can arise when normal glandular changes, such as benign prostatic hyperplasia or cancerous transformation, occur in this region. Meanwhile, the variability in prostate morphology observed during growth and development across different individuals can lead to diverse signal characteristics in the transition zone, further complicating diagnostic interpretations.

Tissue microstructure information is derived from DWI by evaluating the diffusion motion of water molecules in tissues. The apparent diffusion coefficient (ADC) is commonly employed as an indicator to reflect water mobility [13]. Water molecules in normal prostate tissue exhibit relatively unconstrained movement, resulting in intermediate signal intensity on DWI and high signal intensity on ADC maps; whereas, densely packed cancer cells in cancerous tissue impede the diffusion motion of water molecules. Therefore, DWI demonstrates high signal intensity attributable to restricted diffusion, while ADC maps exhibit low signal intensity. An association between the degree of signal reduction on ADC maps and higher Gleason scores has been established [14].

DCE sequences, according to T1-weighted imaging, evaluate tissue hemodynamics through the administration of a contrast agent and the dynamic monitoring of its distribution and metabolism in prostate tissue. Slow and homogeneous enhancement is characteristic of DCE in normal prostate tissue. In comparison, cancerous tissue in DCE exhibits rapid enhancement, displaying a curve represented by a rapid upstroke followed by a rapid washout phase [15]. However, limitations exist in DCE sequences. Benign conditions, such as inflammation or hyperplasia, can also demonstrate early enhancement, leading to the potential for false-positive findings. Studies have indicated that DCE in isolation possesses limited sensitivity and specificity for prostate cancer detection [16]. In addition, the significant expense of contrast agents, along with the potential for allergic reactions, restricts the widespread acceptance of DCE and renders it unsuitable for patients with compromised renal function.

However, the subjective visual assessment in traditional mpMRI interpretation is susceptible to inter-reader variability. Extensive experience is a prerequisite for manual mpMRI interpretation, which is also labor-intensive, and its sensitivity and specificity remain relatively modest, somewhat constraining its clinical utility [17]. Therefore, to minimize discrepancies among readers and variations between radiologists with differing levels of expertise, a more efficient and accurate methodology for mpMRI data interpretation is urgently required.

**Fig 1:**
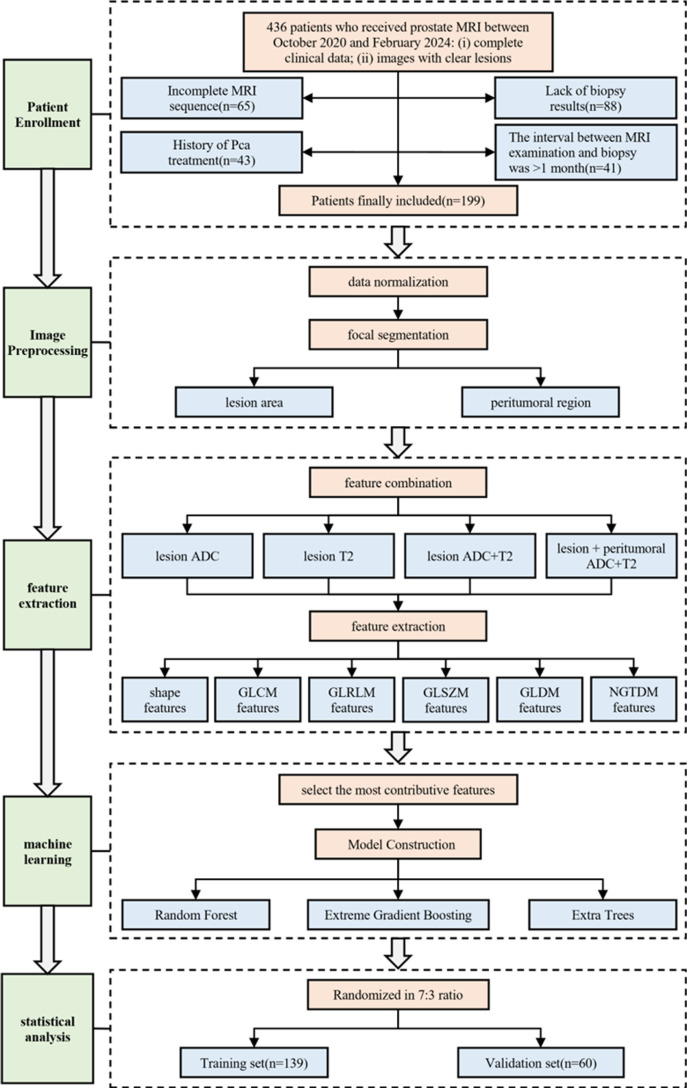
The workflow of this study.

In this study, we conducted a single-center retrospective analysis using multimodal imaging data combined with machine learning techniques to explore methods for diagnosing benign and malignant prostate cancer nodules. Our research not only extracted features from the tumor itself but also examined the impact of peritumoral features on diagnostic models. By analyzing multiparametric MRI (such as T2-weighted imaging and ADC sequences), this study aims to improve the accuracy and stability of prostate nodule diagnosis and address the limitations of traditional imaging methods. This study provides clinicians with a more accurate, non-invasive diagnostic tool, helping reduce unnecessary biopsies and supporting future personalized treatment decisions for prostate cancer. The workflow of this study is shown in Fig 1.

## Literature review

In the field of prostate cancer research, artificial intelligence(AI) and its sub-fields, namely machine learning(ML), radiomics, and deep learning(DL), have made remarkable progress in recent years, while also revealing some limitations[18].

In multiparametric magnetic resonance imaging studies, compared with investigations utilizing single-sequence imaging protocols, JUNG et al. determined that combining T2WI with DWI produces enhanced sensitivity (0.79) and specificity (0.82) in prostate cancer detection, while also improving tumor characterization in the transition zone [19]. High signal intensity and reduced ADC values on DWI are typical presentations of transition zone prostate cancer, which distinguishes it from normal tissue or benign prostatic hyperplasia. Benign prostatic hyperplasia typically exhibits intermediate or mildly high signal intensity on DWI, but its ADC value generally surpasses that of cancerous tissue. Through the application of quantitative analysis of ADC values, DWI can contribute to the differentiation between benign and malignant lesions located in the transition zone. A meta-analysis conducted by HAMOEN et al. has confirmed that mpMRI can achieve a specificity of 0.88 and a sensitivity of 0.74 in the detection of prostate cancer, which is superior to studies employing single sequences [20]. For treatment response monitoring, the integration of information from multiple sequences in mpMRI allows for a more thorough assessment of treatment effectiveness and offers valuable support for clinical decision-making, thereby mitigating the risk of overtreatment [21].

In the domain of AI, the integration of AI-drive image analysis with clinical parameters-including patient age, ethnicity, family history, and lifestyle factors-has substantially enhanced the diagnostic accuracy of prostate cancer detection. This technological advancement demonstrates superior performance compared to conventional diagnostic approaches. Crucially, the implementation of AI enables significant reduction in costly and invasive biopsy procedures, while concurrently reducing financial burdens and shortening turnaround times for diagnostic confirmation. Omran et al. constructed a combined deep-learning model for prostate cancer classification. By leveraging the data of prostate cancer patients who had undergone surgery, they successfully developed a predictive model for clinically significant patient subgroups [22]. This achievement provides a new approach for precise judgment of specific patient groups in clinical practice. However, this model relies on the data of surgical patients, and the data acquisition has certain limitations. Moreover, it does not fully consider the generalization ability of the model under different data sources. Bleker et al. proposed a deep-learning mask (DLM) for the automatic segmentation of the volume of interest (VOI). Research shows that in radiomics based on bi-parametric MRI, the DLM can replace manual segmentation [23]. It enables more accurate and efficient segmentation during the detection of prostate cancer (PCa), thus improving the detection efficiency and accuracy. Mahmood et al. developed a transfer-learning model based on multiparametric MRI. By integrating features extracted from different MRI sequences, they significantly enhanced the accuracy of prostate cancer classification, providing more powerful technical support for disease diagnosis [24]. Zhen Kang and colleagues explored the effectiveness of a deep learning model for classifying and predicting the malignancy of PI-RADS 3 lesions, demonstrating that a ResNet-18-based deep learning model using T2-weighted images could accurately classify PI-RADS 3 lesions [25]. While these AI-based studies suggest that such methods can aid in clinical decision-making, they also have limitations. The quality of MRI images is affected by various factors, such as off-center distortion and imaging parameters, leading to inconsistencies between images obtained from different machines. These differences necessitate image standardization before radiomics analysis, which can introduce errors and impact results [26]. Additionally, most studies rely on data from single institutions, and multicenter studies are rare, making it difficult to compare algorithmic results across different machines and sequence parameters. Furthermore, many studies overlook changes in the peritumoral environment, focusing solely on extracting radiomic features from the tumor lesion for prediction. However, changes in peritumoral radiomics may help predict tumor risk earlier and enable better intervention and treatment [27].

## Method

### Patient inclusion

This study adhered to the Declaration of Helsinki and was approved by the Ethics Committee of Zhejiang Hospital (Ethics Approval Number: 2024 Provisional Review No.117K). As this is a retrospective study that doesn’t involve identifiable personal information, written informed consent from participants was waived. The researchers commit to adhering to Chinese laws and regulations, strictly safeguarding data security and personal privacy. Our article was initially accessed on September 28, 2024, for the purposes of analysis related to our study. We retrospectively collected data from patients who underwent 1.5T multiparametric prostate MRI and subsequent biopsy to confirm the nature of prostate nodules at Zhejiang Hospital between October 2020 and February 2024. The inclusion criteria were: (I) availability of complete clinical data; (II) clearly demarcated lesions visible on all MRI sequences. Exclusion criteria included: (I) a history of prostate cancer (PCa) treatment, including surgery, endocrine therapy, or radiation therapy; (II) the absence of a confirmed PCa diagnosis; (III) incomplete MRI sequences; and (IV) an interval of more than one month between MRI and pathological examination. A total of 436 patients who met the inclusion criteria were initially screened.

### Imaging data collection and annotation

#### Imaging collection.

The imaging data in this study were collected using a 1.5T multiparametric prostate MRI scanner (Siemens MAGNETOM Aera, Siemens Healthcare, Germany). Axial T2-weighted imaging (T2WI) and apparent diffusion coefficient (ADC) imaging were acquired for all patients. T2WI and ADC sequences are widely considered to provide complementary diagnostic information: T2WI excels in evaluating prostate structure and lesion boundary resolution, while ADC helps assess the microdiffusion properties of prostate tissue. The combination of these sequences significantly enhances the accuracy of prostate nodule diagnosis.

The scanning parameters for T2WI and ADC sequences were as follows: T2WI was performed using an axial plane scan with a repetition time (TR) of 3200 ms, echo time (TE) of 90 ms, a slice thickness of 3 mm, a field of view (FOV) of 180 mm, and a matrix size of 256 × 256. For ADC imaging, the b-values ranged from 0 to 1000 s/mm², with a TR of 3500 ms, TE of 70 ms, a slice thickness of 3 mm, and a matching FOV. To ensure image quality, all MRI images were acquired by trained technicians following standardized protocols.

#### Lesion and peritumoral annotation.

For the regions of interest, all imaging data were retrospectively annotated by a radiologist with 10 years of diagnostic experience, using biopsy results as the gold standard for classification [28,29]. Lesion segmentation and annotation were performed using ITK-SNAP (Version 3.8.0), which included both the lesion area and a 3 mm peritumoral region. The inclusion of the peritumoral area was based on existing literature, suggesting that changes in peritumoral tissue may aid in predicting tumor growth and metastasis risk early on. During the annotation process, the radiologist reviewed both the T2WI and ADC sequences, ensuring clear delineation of the lesion boundary and the surrounding tissue structure. To ensure accuracy, another radiologist with 15 years of experience independently reviewed the initial annotations, and any discrepancies were resolved through discussion, resulting in consensus. This annotated data was subsequently used for radiomic feature extraction and machine learning model training, ensuring the quality and consistency of the dataset.

#### Radiomics feature extraction and selection.

In this study, radiomic features were extracted using pyradiomics (Version 3.0), a tool capable of extracting a variety of quantitative features from medical images. For each lesion and its peritumoral region, features were extracted separately from the T2WI and ADC sequences. The specific categories of extracted features included: shape features (such as volume, surface area, etc.), gray level co-occurrence matrix (GLCM) features (such as contrast, entropy, homogeneity, etc.), gray level run length matrix (GLRLM) features (such as long run emphasis, short run emphasis, etc.), gray level size zone matrix (GLSZM) features (such as large area emphasis, small area emphasis, etc.), gray level dependence matrix (GLDM) features, and neighboring gray tone difference matrix (NGTDM) features. To minimize the effects of variability from different scanning conditions, all imaging data were normalized prior to feature extraction.

After feature extraction, we applied a random forest model to select the most contributive features. As an ensemble learning method, random forests can compute the importance score of each feature for classification, helping us identify those most impactful for classification. We performed feature selection for four different combinations: lesion ADC, lesion T2, lesion ADC+T2, and lesion + peritumoral ADC+T2. For each combination, the feature selection process involved training the random forest model and ranking feature importance. By calculating the Gini impurity decrease, the model effectively filtered the most contributive features, which were then used for subsequent classification model construction and validation.

#### Machine learning model construction.

In this study, to further improve the accuracy of classifying benign and malignant prostate nodules, we employed three machine learning algorithms: Random Forest (RF), Extreme Gradient Boosting (XGBoost), and Extra Trees (ET). Each of these algorithms has distinct advantages and is well-suited for handling high-dimensional radiomic features and the complexity of multimodal imaging data.

#### Random Forest (RF).

Random Forest is an ensemble learning method based on decision trees, which determines the final classification result by generating multiple decision trees and using a voting mechanism. In this study, Random Forest was used to process the aforementioned four feature combinations. We used 500 decision trees (n_estimators = 500) to build the model, with a maximum tree depth (max_depth) set at 10 to balance model complexity and generalization capacity.

#### Extreme gradient boosting (XGBoost).

XGBoost is an improved gradient boosting decision tree algorithm, known for its efficient parallel computation and regularization strategies to prevent overfitting. In this study, XGBoost was applied for classification prediction using radiomic features. We employed 100 iterations (n_estimators = 100) and set the learning rate (learning_rate) to 0.05, with a maximum tree depth (max_depth) of 6. To prevent overfitting, we applied L2 regularization (lambda = 1.0) to constrain model complexity and introduced early stopping (early_stopping_rounds = 10) to terminate training automatically if the model began overfitting the data. Additionally, we set the subsample rate (subsample = 0.8) and the feature sampling rate (colsample_bytree = 0.8) to enhance the model’s generalization performance.

#### Extra Trees (ET).

Extra Trees, similar to Random Forest, increases randomness by selecting a split point randomly at each node, rather than calculating the optimal split, thereby enhancing model diversity and generalization capacity. In this study, the ET model was constructed using 500 trees (n_estimators = 500), with a maximum tree depth (max_depth) of 8 and the entropy criterion (entropy) for node splitting.

### Experimental settings

This study trained and validated four feature combinations (lesion ADC group, lesion T2 group, lesion ADC+T2 group, and lesion combined with peritumoral ADC+T2 group). The data was randomly divided into training and validation sets in a ratio of 7:3. Grid search was used for parameter optimization to tune the hyperparameters of random forest, XGBoost, and extreme random tree models (such as the number of decision trees, maximum tree depth, learning rate, etc.). The experimental environment used the Python programming language, based on the scikit-learn and XGBoost libraries, and the calculations were performed on a Linux server equipped with an NVIDIA RTX 3090 graphics card to ensure training efficiency and result stability.

### Statistics

The statistical analysis of this study was mainly used to evaluate the classification performance of each machine learning model under different feature combinations. We used the scikit-learn library in Python for data statistics and model evaluation. For the evaluation of the classification model, a variety of performance indicators were used, including accuracy, recall, precision, F1 value, and area under the curve (AUC). Among them, AUC is calculated by drawing the receiver operating characteristic (ROC) curve to evaluate the classification ability of the model at different decision thresholds. In addition, the SHAP value (Shapley Additive Explanations) is used to explain the contribution of each feature to the model prediction results to help analyze the importance of the feature. The significance level of all statistical tests is set at p < 0.05.

## Results

### Patient enrollment statistics and analysis

**Fig 2:**
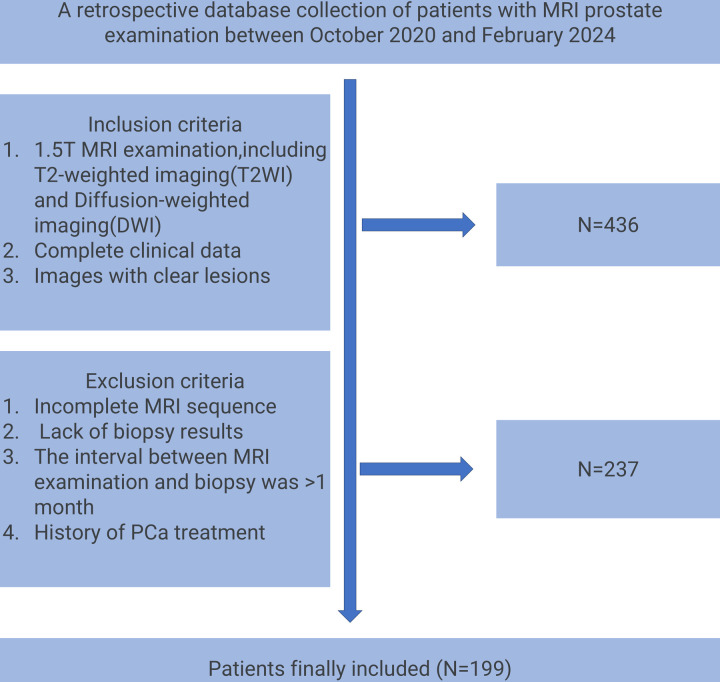
Flow chart of patient enrollment in this study.

This study initially included 436 patients who underwent 1.5T multi-parameter prostate MRI examinations from October 2020 to February 2024. After strict screening of inclusion and exclusion criteria, 199 patients finally met the study conditions. Exclusion criteria included incomplete MRI sequences (n = 65), lack of pathological biopsy results (n = 88), time interval between MRI examination and biopsy exceeding 1 month (n = 41), and history of PCa treatment (n = 43). According to the patients’ MRI results, we further divided these patients into a training group (n = 139) and a validation group (n = 60), including 100 malignant patients and 99 benign patients. MRI data were used for subsequent imaging omics analysis and model construction. The process of patient enrollment is shown in Fig 2. This process not only ensures the integrity and consistency of the data but also provides high-quality baseline data for subsequent image feature extraction and model training. Specifically, the rigorous screening criteria help minimize potential biases introduced by heterogeneous patient populations, thereby enhancing the reproducibility and generalizability of the model. For example, excluding patients with “MRI and biopsy interval of more than one month” effectively avoids the interference of lesion morphological changes caused by time factors on the stability of image features, thereby ensuring the timeliness of feature extraction. Moreover, the inclusion of only well-defined lesions across all MRI sequences strengthens the robustness of radiomic feature analysis, ensuring that extracted features truly reflect the underlying tumor characteristics rather than imaging artifacts.

### Imaging feature extraction and selection results

In this study, we extracted features from MRI images of ADC and T2 modalities, with a total of 988 float-type features extracted from each modality. These features encompass shape, statistical, and texture-related information. Shape features reflect the geometric structure of the lesion, such as volume and surface area; first-order statistical features describe the distribution of pixel intensities, including mean, variance, and skewness. Additionally, texture features include gray level co-occurrence matrix (GLCM) features, which assess spatial relationships between pixel intensities (e.g., contrast, entropy); gray level run length matrix (GLRLM) features, which describe the continuity of pixels with the same intensity; and gray level size zone matrix (GLSZM) features, which measure the size of pixel regions with the same intensity.

To reduce the feature dimensionality, we applied a random forest model for feature selection and ranked the features based on their importance. From each group, we selected the top 50 features contributing most to the classification task (see Supplementary Material 1 for specific feature rankings). In the lesion ADC group, the most important features were concentrated on GLCM’s gray level non-uniformity and short run emphasis. These features reflect local texture variations in the image, which help distinguish between benign and malignant nodules. In the lesion T2 group, the most important features were predominantly shape and GLCM features, with volume shape features and GLCM contrast showing significant importance, reflecting the clarity of lesion boundaries and the homogeneity of internal structures. In the lesion ADC+T2 group, the combined features from both modalities showed higher discriminative power, with features such as gray level variance and small area emphasis in GLSZM displaying high importance. This indicates that combining ADC and T2 modalities provides complementary information, further improving classification accuracy. In the lesion + peritumoral ADC+T2 group, peritumoral features such as GLSZM’s zone variance and GLDM’s dependence variance contributed significantly to the prediction of prostate cancer malignancy. These features capture micro-level changes in the peritumoral tissue, providing more evidence for early tumor detection.

### Machine learning validation results

**Fig 3:**
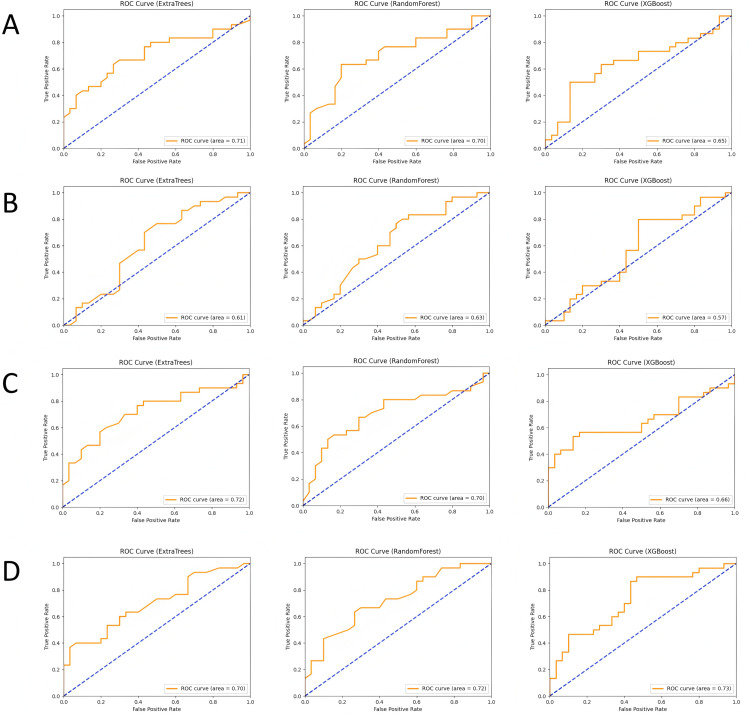
ROC curves and AUC values of each model in different subgroups, where (A-D) represent the lesion ADC group, lesion T2 group, lesion ADC+T2 group, and lesion combined with peritumoral ADC+T2 group, respectively.

**Fig 4:**
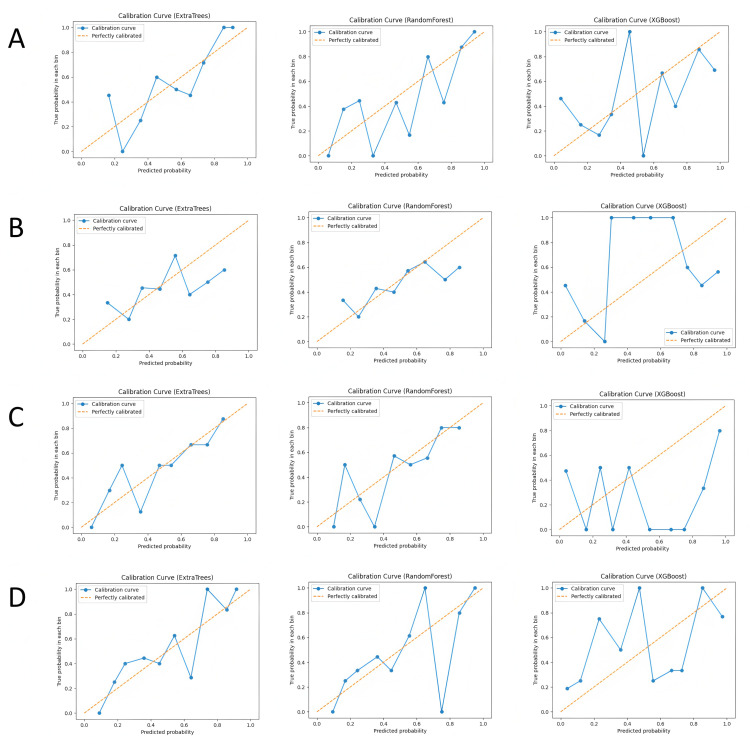
Calibration curves of each model in different subgroups, where (A-D) represent the lesion ADC group, lesion T2 group, lesion ADC+T2 group, and lesion combined with peritumoral ADC+T2 group, respectively.

In this study, we built and validated machine learning models for the four feature combinations (lesion ADC, lesion T2, lesion ADC+T2, and lesion + peritumoral ADC+T2) using three machine learning algorithms: Random Forest (RF), XGBoost, and Extra Trees (ET). The performance of each model was evaluated using metrics such as accuracy, recall, precision, F1-score, and area under the curve (AUC). The ROC curves are shown in Fig 3, calibration curves in Fig 4, and the comparison of evaluation metrics is listed in Table 1.

**Table 1:** Comparison of performance of each model in different subgroups.

Model	Location	Accuracy	Recall	Precision	F1	AUC
RandomForestClassifier	Lesion ADC	0.6333	0.6333	0.6339	0.6329	0.7006
XGBClassifier	Lesion ADC	0.6333	0.6333	0.6333	0.6333	0.6467
ExtraTreesClassifier	Lesion ADC	0.65	0.65	0.6502	0.6499	0.7089
RandomForestClassifier	Lesion T2	0.6167	0.6167	0.62	0.614	0.6278
XGBClassifier	Lesion T2	0.6	0.6	0.6042	0.596	0.57
ExtraTreesClassifier	Lesion T2	0.6	0.6	0.6004	0.5996	0.6144
RandomForestClassifier	LesionT2 + ADC	0.6667	0.6667	0.6667	0.6667	0.6978
XGBClassifier	LesionT2 + ADC	0.5667	0.5667	0.5667	0.5667	0.6567
ExtraTreesClassifier	LesionT2 + ADC	0.6833	0.6833	0.6835	0.6832	0.7211
RandomForestClassifier	Lesion combined with peritumor T2 + ADC	0.6833	0.6833	0.6852	0.6825	0.7222
XGBClassifier	Lesion combined with peritumor T2 + ADC	0.6	0.6	0.6018	0.5982	0.7289
ExtraTreesClassifier	Lesion combined with peritumor T2 + ADC	0.65	0.65	0.6515	0.6491	0.6983

In the lesion ADC group, the Random Forest model demonstrated stable classification performance with an accuracy of 0.633 and an AUC of 0.701. The XGBoost model performed slightly worse, with an AUC of 0.647, while the Extra Trees model achieved the best performance with an AUC of 0.709. This indicates that ADC-based classification can effectively distinguish benign from malignant nodules, especially with the Extra Trees model, which showed stronger classification ability when handling complex features. In the lesion T2 group, the performance of all models was slightly weaker. The Random Forest model achieved an accuracy of 0.617 and an AUC of 0.628, while the XGBoost model had a lower AUC of 0.57, and the Extra Trees model reached an AUC of 0.614. Although T2 features provide some structural information, their contribution to classification is more limited, leading to lower classification performance.

When combining ADC and T2 features, the classification performance improved significantly. The Extra Trees model performed best in this feature combination, with an AUC of 0.721, while the Random Forest model had an AUC of 0.698, and the XGBoost model reached an AUC of 0.657. The complementary nature of ADC and T2 features enhanced classification accuracy, with the Extra Trees model showing superior performance when integrating multimodal data. In the lesion + peritumoral ADC+T2 group, the classification performance reached its highest levels. The Random Forest model had the best performance with an AUC of 0.722, followed by the Extra Trees and XGBoost models with AUCs of 0.698 and 0.729, respectively. The peritumoral features contributed significantly to tumor identification, and integrating this information further improved classification accuracy. By comparing the four feature combinations and three machine learning models, the results suggest that the combination of multimodal imaging data (ADC and T2) and the inclusion of peritumoral features can significantly enhance the accuracy of classifying prostate nodules as benign or malignant.

### Interpretability analysis of image features

**Fig 5:**
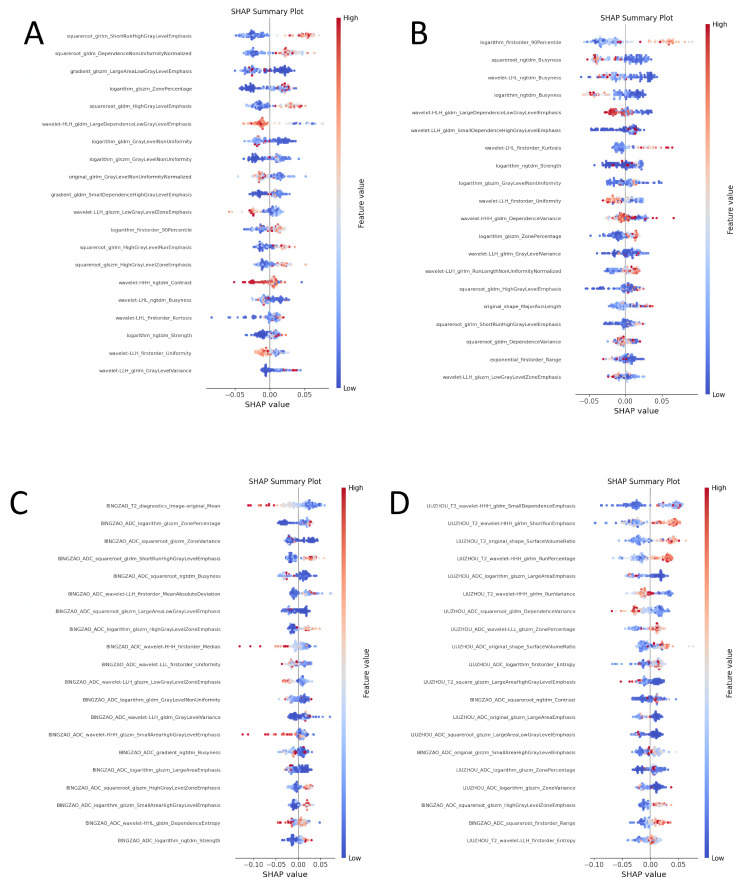
SHAP analysis visualization of the model, where (A-D) represent the lesion ADC group, lesion T2 group, lesion ADC+T2 group, and lesion combined with peritumoral ADC+T2 **group, respectively.**

In order to explore the decision-making basis of each model for the classification of benign and malignant prostate cancer, we used the SHAP (Shapley Additive Explanations) method to perform an interpretative analysis of the machine learning model of four feature combinations (taking random forest as an example). The SHAP value shows the contribution of each feature to the classification result, helping us understand the key features that the model relies on in actual decision-making. The following analysis shows the top 20 most important features in each feature combination and their impact on the classification results (see Fig 5 for visualization).

### Lesion ADC group

In the lesion ADC group, “squareroot_glrlm_ShortRunHighGrayLevelEmphasis” and “squareroot_gldm_DependenceNonUniformityNormalized” became the most important features in the classification. The former measures the emphasis of short-run high gray levels in the image, while the latter relies on a measure of non-uniformity normalization. Both reflect the local gray level changes and texture details in the image. These features contribute significantly to distinguishing benign and malignant nodules. In addition, “gradient_glszm_LargeAreaLowGrayLevelEmphasis” was also shown to play a key role in the classification of malignant tumors. This feature may be related to the spread of malignant tumors. The distribution of SHAP values shows that high eigenvalues (red points) are generally associated with the prediction of malignant tumors, while low eigenvalues (blue points) tend to be associated with benign lesions.

### Lesion T2 group

In the lesion T2 group, “logarithm_firstorder_90Percentile” and “squareroot_ngtdm_Busyness” contribute more to the classification results of the model. “logarithm_firstorder_90Percentile” reflects the distribution of high-intensity pixels in T2 images, indicating the concentration of high gray value pixels, which is of great significance for the identification of malignant lesions. “squareroot_ngtdm_Busyness” describes the busyness of the local area of the image, showing the complexity of the image texture. More complex textures are usually associated with malignant lesions. The high SHAP values (red) of these two features have a positive contribution to the malignant classification, indicating that they are strongly associated with malignant nodules.

### Lesion ADC+T2 group

In the lesion ADC+T2 group, after combining the features of ADC and T2 modalities, the classification ability of the model is further enhanced. SHAP analysis shows that “logarithm_glszm_ZonePercentage” and “squareroot_glszm_ZoneVariance” have a greater impact on classification. “logarithm_glszm_ZonePercentage” measures the proportion of the same grayscale area in the image, while “squareroot_glszm_ZoneVariance” reflects the change in grayscale value within the region. These two features effectively capture the grayscale difference and distribution characteristics of the tumor area, helping the model to better identify malignant lesions. The high and low changes in SHAP values show that the structural complexity of the image area is highly correlated with the malignancy of the tumor.

### Lesion combined with peritumoral ADC+T2 group

In the lesion combined with peritumoral ADC+T2 group, the interpretability of the model is further improved, especially “BINGZAO_ADC_squareroot_glszm_ZoneVariance” and “BINGZAO_ADC_logarithm_glszm_ZonePercentage” become important drivers of classification. Grayscale changes and texture features of the peritumoral region, such as regional variance and regional percentage, can better reveal the diffusive characteristics of malignant lesions. In addition, “BINGZAO_T2_diagnostics_Image-original_Mean”, representing the mean grayscale value of T2 images, showed a significant impact on classification. SHAP value analysis showed that these features were strongly correlated with malignant tumor classification at higher values (red points), while they were consistent with benign classification at lower values (blue points).

## Discussion

This study aimed to construct a classification model for distinguishing between benign and malignant prostate nodules using multimodal imaging data (ADC and T2 sequences) combined with machine learning techniques. We employed radiomic feature extraction and machine learning methods such as random forest to filter and analyze imaging features from both the lesion and peritumoral regions. By validating the models on four feature combinations—lesion ADC, lesion T2, lesion ADC+T2, and lesion + peritumoral ADC+T2—we found that the integration of multimodal imaging and peritumoral feature analysis moderately improved the accuracy of prostate cancer diagnosis.

In recent years, the application of multimodal imaging technology and machine learning in the diagnosis of prostate cancer has made significant progress. For example, the deep learning model proposed by [22] significantly improved the classification accuracy by combining T2 and ADC sequences, but it did not explore the potential value of surrounding area features. [27] verified for the first time the role of surrounding area imaging features in the prediction of early recurrence of liver cancer, providing an important reference for this study. In addition, although the transfer learning framework proposed by [24] performed well on single-modal data, its utilization of multimodal complementarity was still insufficient. In contrast, this study not only improved the classification performance (AUC: 0.729) by integrating multimodal imaging and surrounding area features, but also provided a quantitative basis for early invasion risk assessment, making up for the limitations of existing methods. These advances together support the innovation and clinical significance of this study.

We selected three machine learning algorithms for this study: Random Forest, XGBoost, and Extra Trees, all of which are well-suited for handling high-dimensional, complex data and have robust feature selection capabilities. Random Forest reduces the risk of overfitting by constructing multiple decision trees and using a voting mechanism for classification, while also providing insights into feature importance [30]. In this study, Random Forest demonstrated stable performance in handling multimodal imaging features, especially during the feature selection stage. XGBoost, a gradient-boosting algorithm known for its efficient parallel computing and overfitting prevention, performed slightly worse in some feature combinations but still exhibited strong performance when handling data with strong nonlinear relationships [31]. Extra Trees, which increases model diversity and generalization by randomly selecting split points for features [32], performed the best in this study, achieving the highest AUC in the lesion + peritumoral ADC+T2 group. This indicates that Extra Trees is well-suited for processing multimodal and complementary imaging data, yielding superior classification results.

Through a comparison of different modalities (ADC and T2) and regions (lesion and peritumoral), we found that the fusion of multimodal features and the inclusion of peritumoral features moderately enhanced the accuracy of prostate cancer classification. While ADC and T2 each provide valuable feature information on their own—particularly ADC features, which excel at capturing the diffusion characteristics of the tumor—classification performance is limited when used in isolation. However, the combination of ADC and T2 features (ADC+T2) moderately improved classification performance, especially when the structural and shape information from T2 sequences complemented the texture features from ADC, thereby enhancing the model’s overall ability to recognize the lesion. Furthermore, peritumoral features also played a crucial role in the classification task. Clinically, tumors are not confined to the lesion itself; changes in surrounding tissue often indicate tumor infiltration or spread [33]. We found that including peritumoral imaging features (such as gray-level non-uniformity and dependence variance) further improved the diagnostic accuracy of the model, especially when combined with multimodal features [34]. This finding aligns with clinical experience: micro-level changes in the peritumoral region, especially gray-level and texture irregularities, are often key indicators of malignant tumor spread [35]. Therefore, deep exploration of radiomic features offers the potential for non-invasive assessment of tumor infiltration, compensating for the limitations of traditional imaging evaluations that rely solely on lesion morphology, and further supporting accurate diagnosis of prostate cancer [36].

The clinical significance of this study lies in providing a new non-invasive auxiliary tool for the accurate diagnosis of prostate cancer, particularly excelling in distinguishing between benign and malignant nodules. By combining multimodal imaging data (ADC and T2) with features from multiple regions (both lesion and peritumoral areas), we developed a radiomics-based classification model that offers a more comprehensive assessment of tumor complexity and the extent of its spread. Compared to traditional imaging evaluation methods, this model not only captures morphological and textural features within the tumor but also incorporates imaging information from the peritumoral region, improving the identification of early malignant tumor infiltration, thereby reducing both missed diagnoses and misdiagnoses [37]. Clinically, this non-invasive diagnostic model can assist physicians in the early screening and precise classification of prostate cancer, especially in cases where it is challenging to distinguish nodules using conventional imaging techniques. The application of machine learning technology enhances the efficiency of decision-making, enabling physicians to make accurate judgments in a shorter time, potentially reducing unnecessary biopsy procedures and alleviating the burden on patients [38].

Despite these advantages, there are some limitations. First, this study used retrospective data from a single center, with a relatively small sample size, which may affect the model’s generalizability. In clinical practice, the model’s performance might be influenced by variations in imaging equipment, scanning parameters, and patient populations across different centers. Therefore, future validation with multicenter, large-sample data is required to ensure the stability and applicability of the model. Second, although we combined imaging data from ADC and T2 modalities, other potentially valuable modalities (such as T1 and DWI) were not included in the analysis, which may limit the model’s ability to detect more complex lesions. Finally, although we utilized the SHAP method to provide interpretability for the model, the “black box” nature of machine learning models remains a concern. The lack of transparency, especially in complex machine learning algorithms, may impact physicians’ trust in and acceptance of the model’s predictions in clinical practice.

## Conclusion

By combining multimodal imaging data (ADC and T2) and features from multiple regions (lesion and peritumoral areas), we developed a machine learning-based classification model for distinguishing between benign and malignant prostate nodules and demonstrated its effectiveness in improving diagnostic accuracy and robustness. The results indicate that the complementarity of multimodal imaging features and the inclusion of peritumoral features enhance the model’s classification performance to some extent. This model provides a new technical approach for the non-invasive and accurate diagnosis of prostate cancer, reducing the risk of misdiagnosis and missed diagnoses, and supporting personalized clinical decision-making. However, further validation is needed using multicenter, large-sample data to ensure the model’s broad applicability, and efforts should be made to improve its interpretability to facilitate its adoption in real clinical settings.
